# Neoadjuvant or adjuvant therapy for resectable esophageal cancer: a clinical practice guideline

**DOI:** 10.1186/1471-2407-4-67

**Published:** 2004-09-24

**Authors:** Richard A Malthaner, Rebecca KS Wong, R Bryan Rumble, Lisa Zuraw, members of the Gastrointestinal Cancer Disease Site Group of Cancer Care Ontario's Program in Evidence-based Care

**Affiliations:** 1 University of Western Ontario, London Health Sciences Centre Division of Thoracic Surgery and Surgical Oncology, London, Ontario, Canada; 2 Princess Margaret Hospital, University of Toronto, Toronto, Ontario, Canada; 3 Department of Clinical Epidemiology & Biostatistics, McMaster University, Hamilton, Ontario, Canada

## Abstract

**Background:**

Carcinoma of the esophagus is an aggressive malignancy with an increasing incidence. Its virulence, in terms of symptoms and mortality, justifies a continued search for optimal therapy. A clinical practice guideline was developed based on a systematic review investigating neoadjuvant or adjuvant therapy on resectable thoracic esophageal cancer.

**Methods:**

A systematic review with meta-analysis was developed and clinical recommendations were drafted. External review of the practice guideline report by practitioners in Ontario, Canada was obtained through a mailed survey, and incorporated. Final approval of the practice guideline was obtained from the Practice Guidelines Coordinating Committee.

**Results:**

The systematic review was developed and recommendations were drafted, and the report was mailed to Ontario practitioners for external review. Ninety percent of respondents agreed with both the evidence summary and the draft recommendations, while only 69% approved of the draft recommendations as a practice guideline. Based on the external review, a revised document was created. The revised practice guideline was submitted to the Practice Guidelines Coordinating Committee for review. All 11 members of the PGCC returned ballots. Eight PGCC members approved the practice guideline report as written and three members approved the guideline conditional on specific concerns being addressed. After these recommended changes were made, the final practice guideline report was approved.

**Conclusion:**

In consideration of the systematic review, external review, and subsequent Practice Guidelines Coordinating Committee revision suggestions, and final approval, the Gastrointestinal Cancer Disease Site Group recommends the following:

For adult patients with resectable thoracic esophageal cancer for whom surgery is considered appropriate, surgery alone (i.e., without neoadjuvant or adjuvant therapy) is recommended as the standard practice.

## Background

Carcinoma of the esophagus is an aggressive malignancy with an increasing incidence. Its virulence, in terms of symptoms and mortality, justifies a continued search for optimal therapy. The large and growing number of patients affected, the high mortality rates, the worldwide geographic variation in practice, and the large body of good quality research warrants a clinical practice guideline.

This clinical practice guideline was developed by the Gastrointestinal Cancer Disease Site Group (DSG) of Cancer Care Ontario's Program in Evidence-based Care (PEBC), using the methods of the Practice Guidelines Development Cycle [1]. This practice guideline report is a convenient and up-to-date source of the best available evidence on neoadjuvant or adjuvant therapy for resectable esophageal cancer, developed through systematic review, evidence synthesis, and input from practitioners in Ontario. The PEBC has a formal standardized process to ensure the currency of each clinical practice guideline report. This process consists of the periodic review and evaluation of the scientific literature and, where appropriate, integration of this literature with the original clinical practice guideline information.

The systematic review on neoadjuvant or adjuvant therapy for resectable esophageal cancer, which forms the basis for this clinical practice guideline, is available in a companion document [2]. Based on the systematic review, draft recommendations were developed by consensus of the Gastrointestinal Cancer DSG to create the clinical practice guideline report. The clinical practice guideline is intended to promote evidence-based practice in Ontario, Canada. As part of the PEBC's clinical Practice Guideline Development Cycle, all draft recommendations are sent to Ontario practitioners for external review. The efficacy of this external review process has been previously described [3]. The external review is a mailed survey consisting of items that address the quality of the draft practice guideline report and draft recommendations and whether the draft recommendations should serve as a practice guideline. Final approval of this practice guideline report was obtained from the Practice Guidelines Coordinating Committee (PGCC).

## Methods

### Clinical practice guideline development

#### Systematic review

A systematic review with meta-analysis on neoadjuvant or adjuvant therapy for resectable esophageal cancer was developed by the Gastrointestinal Cancer DSG of Cancer Care Ontario's Program in Evidence-based Care [2]. The evidence examined did not support the use of neoadjuvant or adjuvant chemotherapy or radiotherapy for resectable thoracic esophageal cancer.

### Gastrointestinal cancer disease site group consensus

In discussions regarding the completed systematic review, the Gastrointestinal Cancer DSG agreed that the evidence did not support a recommendation for neoadjuvant or adjuvant chemotherapy or radiotherapy for resectable thoracic esophageal cancer. A recommendation that surgery alone should be the standard of care for this patient population was drafted, and it was recommended that the draft practice guideline be sent out to Ontario practitioners for external review.

The role of radiotherapy alone and chemoradiation alone without surgery is addressed in a separate Gastrointestinal Cancer DSG Clinical Practice Guideline: *Combined modality radiotherapy and chemotherapy in the non-surgical management of localized carcinoma of the esophagus *[4].

## Results

### External review

Practitioner feedback was obtained through a mailed survey of 163 practitioners in Ontario (27 medical oncologists, 21 radiation oncologists, 112 surgeons, and three gastroenterologists). The survey consisted of items evaluating the methods, results, and interpretative summary used to inform the draft recommendations and whether the draft recommendations should be approved as a practice guideline. Written comments were invited. Follow-up reminders were sent at two weeks (post card) and four weeks (complete package mailed again). The Gastrointestinal Cancer DSG reviewed the results of the survey.

Eighty-six surveys (58%) were returned. Twenty-nine respondents (34%) (nine medical oncologists, seven radiation oncologists, and 13 surgeons) indicated that the report was relevant to their clinical practice and completed the survey. Key results of the practitioner feedback survey are summarized below.

1. Number surveyed: 163 practitioners in Ontario, Canada involved in the care of cancer patients

2. Return rate: 58% (mean Gastrointestinal Cancer DSG return rate: 60.2%; range: 51% – 84%)

3. Written comments attached: 10%

4. Agreement with the summary of evidence: 90%

5. Agreement with the recommendation: 90%

6. Approval of the recommendation as a practice guideline: 69%

#### Summary of main findings

Three (10%) respondents provided written comments. One practitioner hypothesized that preoperative chemoradiation might have a role in adenocarcinoma of the lower third of the esophagus (as suggested by Walsh et al [5] with 100% adenocarcinoma and by Urba et al [6] with 75% adenocarcinoma), but not in squamous cell carcinoma (as suggested by Bosset et al [7] and by Le Prise et al [8]). Another respondent noted that the survival advantage at three years for combined treatment for preoperative chemoradiotherapy is discounted in the guideline report, and suggested that the guideline recommend the selection of the option preferred by informed patients. There was a request for an algorithm to help in deciding between surgical and non-surgical treatment. The same respondent commented on the limited discussion on quality of life. Two radiation oncologists disagreed with the recommendations and thought that the draft practice guideline report should not be approved as a practice guideline, but neither provided written comments.

## Discussion

### Gastrointestinal cancer disease site group modifications and actions

After completion of the practitioner feedback survey, additional trials were found. The results of two randomized trials both found surgery alone to be significantly superior to radiation alone [9,10], which resulted in an original draft recommendation regarding radiation alone as a primary modality for localized esophageal cancer being removed from the final practice guideline.

In response to this feedback, the Gastrointestinal Cancer DSG acknowledged that the majority of studies have been performed in squamous cell carcinomas. While adenocarcinomas were included in some studies, a distinction between the two histological subtypes was not made because previous studies have not consistently found that they respond differently to chemotherapy or radiation, and nine references [11-19] were added to support this. The Gastrointestinal Cancer DSG did not feel the evidence was compelling enough to recommend preoperative chemoradiotherapy over surgery alone based on the three-year data. After consideration, the Gastrointestinal Cancer DSG decided not to create an algorithm as suggested as a similar project is currently under development.

After addressing the comments obtained from practitioners during the external review, the Gastrointestinal Cancer DSG voted that the overall guideline recommendations should be approved, and submitted the practice guideline to the Practice Guidelines Coordinating Committee for review.

### Practice guidelines coordinating committee approval process

The practice guideline report was circulated to members of the Practice Guidelines Coordinating Committee for review and approval. All 11 members of the PGCC returned ballots. Eight PGCC members approved the practice guideline report as written and three members approved the guideline conditional on the Gastrointestinal Cancer DSG addressing specific concerns. PGCC members requested that the following issues be addressed prior to the approval of the guideline report:

One member noted that although the majority of studies had been performed in squamous cell carcinomas, some studies included adenocarcinomas, and it would be helpful if the pathological subtypes were discussed. In particular, this member wanted to know if there was any difference in response or outcome for the two histological subtypes. Another member noted that although the pooled analysis for preoperative chemoradiation versus surgery alone detected no difference at one year, the pooled estimate almost reached significance. This member was concerned that the discussion may be too dismissive of the data, and suggested there be some acknowledgment that further follow-up and additional studies are needed.

In response to this feedback, the Gastrointestinal Cancer DSG expanded on the earlier revisions concerning the similarities in response to treatment between squamous cell carcinomas and adenocarcinomas.

Also, after the original practice guideline was submitted to the PGCC, two meta-analyses [20,21] both detecting a statistically significant difference in survival at three years favouring preoperative chemoradiation versus surgery alone were obtained. The Gastrointestinal Cancer DSG re-pooled the mortality data from the six trials [5-8,22,23] at three years and obtained similar results.

## Conclusions

In consideration of the systematic review, external review, and subsequent Practice Guidelines Coordinating Committee revision suggestions, and final approval, the Gastrointestinal Cancer Disease Site Group developed the following Clinical Practice Guideline:

### Practice guideline

This practice guideline reflects the most current information reviewed by the Gastrointestinal Cancer DSG.

#### Target population

These recommendations apply to adult patients with resectable and potentially curable thoracic (lower two-thirds of esophagus) esophageal cancer for whom surgery is considered appropriate.

#### Recommendation

• If surgery is considered appropriate, then surgery alone (i.e., without neoadjuvant or adjuvant therapy) is recommended as the standard practice for resectable thoracic esophageal cancer.

This Clinical Practice Guideline report is based on work completed in October, 2003. All approved PEBC Clinical Practice Guideline reports are updated regularly. Please see the PEBC's web site  for a complete list of current and on-going projects.

## Competing interests

The author(s) declare that they have no competing interests.

## List of abbreviations used

In order of appearance:

DSG, Disease Site Group; PEBC, Program in Evidence-based Care; PGCC, Practice Guidelines Coordinating Committee.

## Authors' contributions

RM, RW, and LZ created the initial drafts of this clinical practice guideline with input from other members of the Gastrointestinal Cancer DSG. RM, RW, and BR created the final draft of this clinical practice guideline. Creation of the submitted manuscript was performed by BR and RM.

## Pre-publication history

The pre-publication history for this paper can be accessed here:


